# Electrical stimulation: a missing key to promote maturation of human pluripotent stem cell-derived cardiomyocytes in three-dimensional cardiac tissues

**DOI:** 10.3389/fbioe.2025.1686342

**Published:** 2025-10-09

**Authors:** Sem Sterckel, Robert Passier, José Manuel Rivera-Arbelaez

**Affiliations:** ^1^ Applied Stem Cell Technologies, Department of BioEngineering Technologies, Cardiovascular Health Technology Centre, TechMed Centre, University of Twente, Enschede, Netherlands; ^2^ Department of Anatomy and Embryology, Leiden University Medical Centre, Leiden, Netherlands; ^3^ BIOS Lab-on-a-Chip Group, MESA+ Institute for Nanotechnology, University of Twente, Enschede, Netherlands

**Keywords:** hiPSC-CM maturation, organ-on-a-chip, electrical stimulation, engineered heart tissue, bioelectrical cues, cardiac tissue engineering, 3D cardiac tissue

## Abstract

The maturation of human pluripotent stem cell-derived cardiomyocytes (hPSC-CMs) remains a major challenge in developing functional *in vitro* cardiac models. While three-dimensional (3D) culture systems improve structural and metabolic properties, they do not fully recapitulate adult cardiomyocyte physiology. Exogenous electrical stimulation has emerged as complementary strategy to further drive maturation. This review highlights both the maturation effects of electrical pacing in 3D cardiac tissues, including enhanced sarcomere organization, conduction velocity, calcium handling, and contractile function. It also discusses the technological parameters used to achieve these outcomes, such as electric field voltage (EFV), pulse duration (PD), stimulation waveform, electrode materials, and pacing protocols, and how these factors influence hPSC-CM development. Despite progress, further research is needed to optimize stimulation setups and to integrate electrical pacing with other maturation cues. Advancing high-throughput, miniaturized platforms will be essential for translating these models into biomedical applications like drug discovery and disease modeling.

## 1 Introduction

The development of protocols for differentiating cardiomyocytes from human pluripotent stem cells (hPSC-CMs) has revolutionized cardiovascular research. hPSC-CMs offer novel opportunities for disease modeling, evaluating the efficacy of therapeutic agents, predicting drug-induced cardiotoxicity, developing new therapeutics and advancing regenerative medicine (Andrysiak et al., 2021; Jebran et al., 2025; Stein et al., 2021).

However, hPSC-CMs exhibit an immature phenotype that closely resembles early fetal cardiomyocytes rather than mature cardiomyocytes of an adult human heart. To address this, researchers have explored various strategies to enhance the maturation of hPSC-CMs, including co-culture systems, biochemical stimuli, and topological cues (Ahmed et al., 2020; Yang et al., 2014). Among these, three-dimensional (3D) culture systems have shown particular promise in promoting maturation (Eschenhagen et al., 2012; Yang et al., 2014). Unlike traditional two-dimensional (2D) cultures, 3D systems better mimic the physiological environment of the myocardium by allowing organized cell-cell and cell-extracellular matrix (ECM) interactions, which are essential for maturation. Specifically, 3D scaffolds or self-assembled tissues enable cells to form intercellular junctions (e.g., Connexin 43-rich gap junctions) and integrin-mediated cell-ECM adhesions in all three dimensions, processes that are significantly restricted in flat 2D monolayers. Culturing hPSC-CMs in 3D systems promotes superior structural and metabolic maturation, with improved myofibrillar alignment, enhanced electrophysiological properties such as sodium current density and action potential upstroke velocity, and a metabolic shift toward oxidative phosphorylation (Lemoine et al., 2017; Ulmer et al., 2018).

Nevertheless, the 3D configuration still does not fully resemble the physiology of the *in vivo* situation. Therefore, electrical stimulation has emerged as complementary strategy to promote maturation of hPSC-CMs, given the critical role of electromechanical excitability in CMs. In the adult heart, CMs rely on electrical depolarization initiated by pacemaker cells rather than self-excitation (Scuderi and Butcher, 2017; Stoppel et al., 2016). In contrast, immature hPSC-CMs typically exhibit spontaneous excitation. Studies suggest that exogenous electrical stimulation can mimic the physiological excitation provided by neighboring cells, thereby promoting both structural and electrophysiological maturation (da Silva et al., 2020; Stoppel et al., 2016). Applying electrical stimuli in 3D culture systems requires specialized bioreactors and bioprinted scaffolds (Licata et al., 2025b; Ronaldson-Bouchard et al., 2018). The transition from 2D to 3D introduces a new level of complexity, as the larger dimensions and volumes alter the electrical field and current density. Consequently, the optimal electrical stimulation parameters identified in 2D models may not be directly translatable to 3D systems, highlighting the need for careful re-evaluation and optimization.

Recent trends indicate that combining multiple maturation strategies can further accelerate the development of mature hPSC-CMs (Ahmed et al., 2020; Li et al., 2025; Stein et al., 2021). This review focuses on the intersection of electrical stimulation and 3D culture systems, providing an overview of key advancements in this multidisciplinary field. We aim to highlight the most promising strategies for enhancing cellular maturation using electrical stimulation on 3D culture systems, discuss potential limitations, and offer insights into future directions for research in the field of hPSC-CM maturation.

## 2 Engineered hPSC-CM maturation

In this section, we explore the biological maturation of hPSC-CMs achieved through electrical stimulation, focusing on improvements in morphology, gene and protein expression, electrophysiological characteristics, calcium handling, contraction, and pharmacological responsiveness. Table 1 summarizes relevant advancements in hPSC-CM maturation reported in studies that directly compare electrically stimulated 3D culture systems with non-stimulated counterparts. Collectively, these studies demonstrate that electrical stimulation is a powerful driver of maturation across multiple biological domains.

**TABLE 1:** Three-dimensional hPSC cardiac models and the improved cardiac characteristics induced by exogeneous electrical stimulation.

Author	Model	Functional			Biological	
		Action potential	Calcium handling	Other	Expression	Morphology
Chan et al. (2013)	EB (hESC-CM)	Increase APD50/APD90>=0.8 (ES: 48% vs. non-ES: 29%) Increase APD90 < 230 ms (ES: 39% vs. non-ES: 59%)	Increase amplitude of calcium transients Shortened calcium release and decay rates	More physiological caffeine response	Gene expression: +MLC2v, +SCN5A, +SERCA, +Kv4.3 Protein expression: + % positive troponin T cells	Cell elongation
Hernández et al. (2016)	EB (hiPSC)	Spontaneous beating EBs (ES: 11.7% ± 2.3% vs. non-ES: 4.9% ± 1.9%) Baseline field potential duration (ES: 0.30 ± 0.19 s vs. non-ES 0.10 ± 0.02 s)	–	Responsiveness to isoproterenol and carbamylcholine	Gene expression: ** (temporarily, 7 days after stimulation) +NKX2.5, +TBX5, +ACTC1, +TNNT2, +MYH7, +MYL7, and increased MYL2/MYL7 ratio	-
Hirt et al. (2014)	EHT Hanging Pillars (hiPSC-CM)	–	–	FC (ES: 0.083 mN VS non-ES: 0.056 mN)	–	Longer and less round CM with higher cytoplasm-to-nucleus ratio Longitudinally oriented CM, more stringently aligned CM, clearer cross-striation, more contractile mass relative to cell-free matrix
LaBarge et al. (2019)	Spheroid (hiPSC-CM)	Beating rate (ES: 70.7 ± 3.8 bpm VS non-ES: 46.4 ± 1.0 bpm) APD50 (ES: 181 ± 67 ms VS non-ES: 217 ± 69 ms) CV (ES: 6.0 ± 0.9 cm/s VS non-ES: 7.8 ± 2.5 cm/s)	Minimum pacing cycle length (ES: 338 ± 21 ms VS non-ES: 569 ± 92 ms)	–	Gene expression: +Cx43, +N-Cad, +MLC2v, +cTnT, +cTnI, - MLC2a Protein expression: +CX43, +N-Cad, +MLC2v, +cTnT	Improved Z-band structure and mitochondrial arrangement
Licata et al. (2025a)	EB (hiPSC-CM)	–	Altered calcium transient: 10% and 50% peak width	–	Gene expression: Altered MYL2, MYH6, IRX4, KCNA5 Protein expression: Altered % of MLC2v, and MLC2a positive cells	-
Nunes et al. (2013)	Biowire (hiPSC-CM)	ET (ES: 1.5 V/cm VS non-ES: 3 V/cm)* CV (ES: 15 cm/s VS non-ES: 10 cm/s)*	MCR (ES: 4 Hz VS non-ES: 3 Hz)* Faster rising slope and time to peak Faster tau decay and time to base	Responsiveness to caffeine	–	Organized sarcomeric banding, converging myofibrils, aligned z discs, mitochondria positioned closer to contractile apparatus, higher presence of H zones per sarcomere, higher presence of I bands per Z disc, higher number of desmosomes per membrane length
Richards et al. (2016)	Spheroid (hiPSC-CM)	Spontaneous beat rate (ES:25.6 ± 2.8 bpm VS non-ES: 29.2 ± 3.9 bpm)	–	–	Protein expression: +Cx43, +N-cad, +MYL2, +MLC-2v Gene expression: -HCN4	-
Ronaldson-Bouchard et al. (2018)	EHT Hanging Pillars (hiPSC-CM: hdFB–75%: 25%)	Resting membrane potential (ES: −70 ± 2.7 mV VS non-ES: 55 ± 5 mV*) CV (ES:25 ± 0.9 cm/s VS non-ES: 10 ± 2 cm/s*)	More mature functional intracellular calcium stores	Responsiveness to caffeine and isoproterenol Positive FFR Positive FDAR Positive PRP	Gene expression: Changes towards more adult like*** (heatmap), increased MLC2v/MLC2a ratio, +ATP2A2, +SLC8A1 Protein expression: +Cx43	Cell size was increased, cell elongation, percent area increase of mitochondria Orderly sarcomeres, mitochondria positioned adjacent to the contractile apparatus, increased sarcomere length, T-tubules colocalized with BIN1, RYR2, Cav1.2 expression, and presence of I-bands, A-bands, M lines, Z lines, desmosomes, and intercalated discs
Ruan et al. (2016)	EHT Standing Pillars (hiPSC-CM)	–	–	Contractility (ES: 1.34 ± 0.19 mN/mm2 VS non-ES: 0.63 ± 0.10 mN/mm 2) Less negative FFR	Protein expression: Slight increase in SERCA2 and RYR2 (non-sign)	
Thavandiran et al. (2013)	EHT Standing Pillar (hESC-CM: FB–75%: 25%)	–	MCR (ES: 6.25 Hz aprox VS non-ES: 5 Hz)*	–	–	–
Xiao et al. (2014)	Biowire (hESC-CM)	ET (ES: 3.5 V/cm VS non-ES: 5.5 V/cm)*	MCR (ES:2.25 Hz VS non-ES: 1.75 Hz)*	–	–	–
Zhang et al. (2023)	EHT Standing Pillars (hiPSC-CM: hcFB–90%: 10%)	–	Shortened calcium release and decay rates Time to peak (ES:190 ± 20 ms VS non-ES 230 ± 20 ms)* Time from peak 50% (ES:200 ± 20 ms VS non-ES:300 ± 20 ms)*	Normalized force by cross-sectional area (non-ES: 29uN/mm2 vs. ES: 36uN/mm2)* More physiological isoprenaline response	Gene expression: +CX43	Anisotropy of sarcomeres, directional alignment

*Value estimated from graph. **Data only significant in 1 of 2 tested cell lines. ES, Electrical stimulation; EB, Embryoid Bodies; EHT, Engineered Heart Tissue; hiPSC-CM, human induced Pluripotent StemCell derived Cardiomyocyte; hd, human dermal; hc, human cardiac; FB, Fibroblast; FC, contraction force; FFR, force-frequency relationship; ET, excitation threshold; MCR, maximum capture rate; APD, action potential duration; FDAR, frequency-dependent acceleration of relaxation; CV, conduction velocity.

### 2.1 Tissue culture systems

The 3D culture systems employed in the studies to enhance maturation of CMs include scaffold-free systems, such as embryoid bodies (EBs) and spheroids, as well as scaffolded systems like engineered heart tissues (EHTs). Scaffold-free systems are inherently cost-effective and straightforward to generate using standard 96 well plate platforms, making them particularly well-suited for applications like drug screening (Campostrini et al., 2021). In contrast, scaffolded systems offer enhanced cell alignment due to the guided tissue organization, and EHTs specifically enable the measurement of absolute contractile force, a key functional readout. However, a limitation of the scaffolded systems is that they often require custom fabrication, such as microfabricated chips, bioreactors, or specialized holders compatible with multiwell platforms.

In scaffolded systems like EHTs, tissue formation can benefit from incorporating a defined proportion of fibroblasts (Campostrini et al., 2021). In some studies, co-culturing with fibroblasts is considered essential for the formation of functional cardiac tissue, as specific fibroblast ratios have been shown to improve electrophysiological characteristics, cellular organization, and force generation (Thavandiran et al., 2013; Tiburcy et al., 2017; Zhao et al., 2020). In the adult heart, fibroblasts are major contributors to the extracellular matrix, providing mechanical and structural integrity (Scuderi and Butcher, 2017; Stoppel et al., 2016). Furthermore, co-culture models incorporating smooth muscle and endothelial cells have further enhanced contractile performance and electrophysiological properties of engineered cardiac tissues (Cofiño-Fabres et al., 2024; Giacomelli et al., 2017). In addition, Giacomelli et al., 2020 demonstrated that cardiac tissue maturation is further enhanced through tri-cellular crosstalk between cardiomyocytes, fibroblasts, and endothelial cells through cAMP signaling. Notably, only cardiac fibroblasts promote structural, functional, and electrophysiological maturation via connexin-43 gap junctions, in contrast to dermal fibroblasts.

### 2.2 Tissue and cell morphology

The adult CM phenotype is characterized by large, rod-shaped, often multinucleated cells with well-organized, elongated sarcomeres and aligned contractile filaments (Denning et al., 2016; Hong et al., 2023). 3D tissue culture systems enhance CM maturation by displaying an increase in cell size, elongation, and notably, sarcomere organization and alignment. The addition of electrical stimulation further promotes maturation of cell shape and size toward the adult CM phenotype (Chan et al., 2013; Hirt et al., 2014; Ronaldson-Bouchard et al., 2018). Enhanced sarcomere organization and alignment resulting from electrical stimulation have been reported (see Table 1), highlighting the synergistic effect of these two maturation strategies.

The well-organized structure of sarcomeres is distinguished by the presence of I-, H-, A-, M-bands, and Z-discs, each formed through the precise alignment of cytoskeletal proteins such as actin, myosin, and titin (Denning et al., 2016). Most hPSC-CM differentiation protocols typically exhibit only Z-discs and M-bands. However, Ronaldson-Bouchard et al. and Nunes et al. observed respectively additional I-, A-, M-, and Z-bands and H-, Z-, and I-bands in electrically stimulated hPSC-CM tissues. This higher organization is associated with increased contractile force normalized to tissue cross-sectional area.

Another hallmark of maturation is the localization of specific structures. Mitochondria, for instance, are abundant in adult cardiomyocytes to meet high energy demands (Denning et al., 2016). In immature hPSC-CMs, however, they are fewer and clustered around the nucleus. Electrical stimulation has been shown to improve mitochondrial distribution with LaBarge et al. reporting enhanced organization and Nunes et al. and Ronaldson-Bouchard et al. reporting mitochondria positioned closer to the contractile apparatus.

Desmosomes, crucial for cardiomyocyte adhesion, are absent in immature hPSC-CMs (Hong et al., 2023). However, both Nunes et al. and Ronaldson-Bouchard et al. reported the presence of desmosomes in electrically stimulated tissues. T-tubules, which are also rarely observed in hPSC-CMs (Hong et al., 2023), were detected by Ronaldson-Bouchard et al. following electrical stimulation. The presence of these structures indicates improved tissue organization and suggests enhanced excitation-contraction coupling (Denning et al., 2016; Hong et al., 2023).

### 2.3 Gene and protein expression

Gene and protein expression analysis provide important insights into the maturation level of electrically stimulated hPSC-CMs. Electrical stimulation elevates the expression of genes encoding contractile proteins like TNNT2 and MYH7, reflecting enhanced assembly of the contractile apparatus (LaBarge et al., 2019; Richards et al., 2016). Furthermore, cell-cell junction proteins, including Cx43 and N-cadherin, are upregulated, underscoring their roles in electrical and mechanical coupling of CMs (LaBarge et al., 2019; Richards et al., 2016; Ronaldson-Bouchard et al., 2018; Zhang et al., 2023). The localization of these proteins at intercalated discs is crucial for maintaining functional syncytia in adult heart tissue (Scuderi and Butcher, 2017).

Proteins involved in calcium handling, such as ATP2A2 (SERCA2), RYR2, and Cav1.2, are also upregulated (Ronaldson-Bouchard et al., 2018), indicating improved calcium cycling and excitation-contraction coupling (Scuderi and Butcher, 2017). The co-localization of T-tubules with calcium-handling proteins like BIN1, RYR2, and Cav1.2 further supports the presence of more efficient and mature calcium dynamics (Ronaldson-Bouchard et al., 2018). In parallel, the downregulation of HCN4 (a marker of pacemaker-like activity), along with an increased MLC2v/MLC2a ratio, indicates a shift toward a more ventricular-like and electrically stable, mature CM phenotype (Richards et al., 2016; Licata et al., 2025a) further showed that the timing of stimulation can bias cardiomyocytes toward atrial- or ventricular-like phenotypes, as reflected in shifts in *MYL2*, *MYH6*, *IRX4*, *KCNA5* expression and the MLC2v/MLC2a positive cell ratio. The potential to steer cardiomyocyte subtypes, such as ventricular, atrial, or nodal, through electrical stimulation has been suggested before, but further research is required to distinguish this mechanism from generic cardiomyocyte maturation (Ma et al., 2018).

In studies aiming to steer early differentiation towards a cardiac fate, Hernández et al. reported a transient upregulation of early cardiac differentiation markers, such as *GATA4*, *NKX*2.5, and *TBX5*, on day 7, but not on day 14 after electrical stimulation. Notably, this effect was only obtained in one of two tested cell lines. Furthermore, Chan et al. observed an increase in troponin-positive cells after stimulation, but the overall yield remained low (<20%) compared to state-of-the-art protocols that routinely achieve >80% (Ronaldson-Bouchard et al., 2018). Taken together, these findings suggest that, in the forms tested, electrical stimulation shows limited robustness and efficiency in directing early cardiac differentiation.

### 2.4 Electrophysiological maturity and conduction velocity

In terms of electrophysiological characteristics, adult CMs differ from the relatively immature hPSC-CMs. For example, the conduction velocity (CV) of adult CMs is approximately 60 cm/s, while hPSC-CMs generally exhibit slower velocities, 10–20 cm/s (Ahmed et al., 2020). Electrical stimulation has been shown to enhance CV, as reported by Ronaldson-Bouchard et al. and Nunes et al., who observed increases from 10 cm/s to 25 cm/s and 10 cm/s to 15 cm/s, respectively, compared to non-stimulated tissues. However, findings are not entirely consistent: Ruan et al. did not observe a significant change in CV with stimulation, and Labarge et al. even reported a decrease in CV. The improvement in CV is often associated with the enhanced expression and localization of cell-cell junction proteins, such as Cx43 and N-cadherin at intercalated discs, which improve electrical coupling. The lower CV in Labarge et al.’s study might be attributed to the use of spheroids as opposed to scaffolded structures, which generally exhibit less structural organization than scaffolded tissue constructs.

### 2.5 Action potential characteristics

An in-depth characterization of the action potential provides key insights into the repolarization kinetics, refractory periods, and overall electrophysiological maturity of hPSC-CMs. Chan et al. observed a greater number of cells with APD50/APD90 ratios above 0.8, a characteristic more closely resembling the action potential of mature ventricular CMs. Additionally, Ronaldson-Bouchard et al. reported lower resting membrane potentials of −70 mV in electrically stimulated tissues, closer to the −80 to −90 mV typical of mature CMs (Denning et al., 2016). These parameters are closely linked to the functioning of ion channels such as sodium, potassium, and calcium channels, which play crucial roles in shaping the action potential. For example, the excitation threshold (ET), which reflects the readiness of sodium channels to start depolarization in response to stimuli, was lowered in the electrically stimulated tissues (Nunes et al., 2013; Xiao et al., 2014).

### 2.6 Calcium handling and excitation-contraction coupling

The analysis of calcium kinetics through calcium imaging provides critical insights into calcium handling maturity in hPSC-CMs. Time to peak, peak-to-base, and decay rates indicate improved calcium reuptake and extrusion due to continuous electrical stimulation (see Table 1), which are essential for efficient excitation-contraction coupling. This improvement is likely caused by the presence of T-tubules and calcium-handling proteins like BIN1 and RYR2, both of which were found to be upregulated in response to continuous electrical stimulation (Ronaldson-Bouchard et al., 2018). These advancements in calcium handling help reduce the risk of arrhythmias, a major concern in immature cardiac models. In addition, calcium transient morphology can shift toward chamber-specific phenotypes depending on stimulation protocol. For instance, alterations in 10% and 50% peak width have been reported, consistent with steering cardiomyocytes toward either more atrial- or ventricular-like calcium handling profiles (Licata et al., 2025a). Testing the maximum capture rate (MCR), the highest pacing frequency a tissue can follow, can further evaluate the functional maturity of hPSC-CMs. Continuous electrically stimulated tissues have demonstrated higher MCRs, indicating an improved ability to adhere to higher pacing frequencies compared to unstimulated tissues (LaBarge et al., 2019; Nunes et al., 2013; Thavandiran et al., 2013).

### 2.7 Contraction force and contraction time curves

Mature CMs typically exhibit higher contraction forces relative to their cross-sectional area, largely due to a more developed and well-aligned contractile apparatus (Scuderi and Butcher, 2017). Electrical stimulation has been shown to increase force generation by approximately 112%, 48%, and 24%, as demonstrated by Ruan et al., Hirt et al., and Zhang et al. respectively. This increase can be attributed to improved cellular alignment and sarcomere organization within the tissue.

To assess the combinatory functioning of the excitation-contraction coupling and calcium handling in hPSC-CMs, multiple phenomena can be studied. A hallmark of mature CMs is a positive force-frequency relationship (FFR), where the contraction force increases with higher pacing frequencies due to more efficient calcium cycling. This positive FFR was observed by Ronaldson-Bouchard et al. while a less negative FFR was observed by Ruan et al., suggesting that electrical stimulation promotes this hallmark of maturity. Notably, a positive FFR has also been reported in electrically paced EHTs by Tamargo et al. Similarly, frequency-dependent acceleration of relaxation (FDAR) is an indicator of enhanced calcium reuptake by SERCA2, enabling faster relaxation at higher pacing frequencies, which was reported by Ronaldson-Bouchard et al. in electrically stimulated tissues. Another important indicator of calcium handling maturity is post-rest potentiation (PRP), which reflects the heart’s ability to increase contraction force following a period of rest due to improved calcium storage in the sarcoplasmic reticulum. Ronaldson-Bouchard et al. observed significant PRP in their electrically stimulated tissues, further demonstrating enhanced calcium cycling and overall tissue maturity. Together, these combinatory features, positive FFR, FDAR, and PRP, provide strong functional evidence of advanced calcium handling and excitation–contraction coupling in electrically matured hPSC-CM tissues.

### 2.8 Pharmacological testing of functional maturity

To assess the functional maturity of these cardiac *in vitro* models in a manner aligned with their intended future applications, such as drug screening, disease modeling, and mechanistic studies, it is crucial to evaluate their responsiveness to pharmacological agents. For example, beta-adrenergic agonists like isoproterenol were used to simulate sympathetic stimulation, which resulted in an increased beating rate and contractile force (Hernández et al., 2016; Ronaldson-Bouchard et al., 2018; Zhang et al., 2023). The observation that electrically stimulated hPSC-CMs respond significantly more intense to these agents, in contrast to non-stimulated controls, highlights the enhanced receptor-mediated signaling pathways induced by electrical stimulation.

Similarly, cholinergic agonists such as carbamylcholine are used to evaluate the response to parasympathetic stimulation via M2 receptor-mediated signaling. Interestingly, a reduction in beating rate was observed by Hernández et al. after carbamylcholine stimulation in the electrically stimulated tissues and not in control tissues. Additionally, drugs like caffeine, which promote calcium release from the sarcoplasmic reticulum, are frequently used to assess calcium handling in these models (Chan et al., 2013; Nunes et al., 2013; Ronaldson-Bouchard et al., 2018). The improved physiological responses to these pharmacological agents in electrically stimulated tissues compared to non-stimulated tissues underscore their increased functional capacity and predictive value. This approach is critical to ensure that hPSC-CM tissues can reliably recapitulate adult cardiac physiology for future applications in drug testing and disease modeling.

## 3 Electrical engineering setup

Having established the relevance of electrical stimulation in enhancing the physiological and morphological maturation of hPSC-CMs, it is important to evaluate the technological parameters driving these improvements. Variables such as electric field voltage (EFV), pulse duration, waveform, electrode material and stimulation frequency can all affect the culture microenvironment. Table 2, summarizes the key technological parameters employed in the previously discussed studies that compared maturation outcomes in 3D hPSC-CM *in vitro* models. By understanding how these parameters contribute, both individually and collectively, we can better elucidate the mechanisms underlying electrical maturation and further optimize stimulation protocols to maximize their efficacy.

**TABLE 2:** Electrical stimulation setup characteristics and parameters used for three dimensional cardiac tissue maturation.

Author	Model	Pacing device	Electrode material	Electrical field parameters	Waveform	Pacing timeframe
Hernández et al. (2016)	Embryoid Bodies (EB)	Custom made	Platinum (Pt) coated gold electrodes	EFV: 2 V/cm PD: 1 ms per polarity Freq: 1 Hz	Polarity: Biphasic Shape: ND Symmetry: Symmetric	Start: After 5 days of EB formation Duration: 5 min
Hirt et al. (2014)	Hanging Pillar EHT	S88X, Grass	Carbon electrodes	EFV: 2 V/cm PD: 4 ms (2 ms in both polarities) Freq: Varying Hz	Polarity: Biphasic Shape: Rectangular Symmetry: Symmetric	Start: After 4 days of tissue formation (day of spontaneous beating) Duration: 10 days Frequency: 2 Hz first week, after 1.5 Hz
LaBarge et al., 2019	Spheroids	C-Pace Unit, IonOptix	Carbon electrodes	EFV: 6.5 V/cm PD: 5 ms Freq: 2 Hz	Polarity: Biphasic Shape: Rectangular Symmetry: Symmetric	Start: After day 7 of spheroid formation Duration: 7 days
Licata et al. (2025b)	EB	Custom made	Stainless stell	EFV: 5 V/cm PD: 5 ms Freq: 1 or 3 Hz	Polarity: Monophasic Shape: Rectangular	Start: After 3, 7, 10, or 14 days of EB formation Duration: 5 days
Nunes et al. (2013)	Biowire	S88X, Grass	Carbon electrodes	EFV: 3–4 V/cm PD: 1 ms Freq: Varying Hz	Polarity: Biphasic Shape: Rectangular Symmetry: Symmetric	Start: After 1 week of tissue formation Duration: 7 days Frequency: Starting at 1 Hz increasing with 0.83 Hz daily until 6 Hz
Richards et al. (2016)	Spheroids in between wires	C-Pace Unit, IonOptix	Carbon electrodes with conductive silicon nanowires	EFV: 2.5 V/cm PD: 5 ms Freq: Varying Hz	Polarity: ND Shape: ND Symmetry: ND	Start: After 9 days of spheroid formation Duration: 9 days Frequency: Starting at 0.5 Hz on day 10, 0.75 Hz- day 11, 1 Hz onwards
Ronaldson-Bouchard et al. (2018)	Hanging pillar EHT	S88X, Grass	Carbon electrodes (perpendicular along axis of tissue)	EFV: 3.5–4V/cm PD: 2 ms Freq: Varying Hz	Polarity: Monophasic Shape: Rectangular	Start: After 7 days of tissue formation Duration: 3 weeks Frequency: Starting at 2 Hz on day 7 to 6 Hz on day 21 with increments of 0.33 Hz. Then 2 Hz until day 28
Ruan et al. (2016)	Standing Pillar EHT (with static stress)	C-Pace EP Culture Pacer, IonOptix	Carbon electrodes	EFV: 5 V/cm PD: 5 ms Freq: 2 Hz	Polarity: ND Shape: ND Symmetry: ND	Start: After 1 week of tissue formation Duration: 1 week
Thavandiran et al. (2013)	Standing Pillar EHT	S88X, Grass	0.005 inch Pt electrodes	EFV: 6 V/cm PD: 1 ms Freq: 1 Hz	Polarity: Biphasic Shape: Rectangular Symmetry: ND	Start: After 72 h of cultivation Duration: 4 days
Xiao et al. (2014)	Biowire	S88X, Grass	Carbon electrodes	EFV: 3.5–4 V/cm PD: 1 ms Freq: 1 Hz	Polarity: Biphasic Shape: Rectangular Symmetry: Symmetric	Start: After 4 days of tissue formation (spontaneous beating was synchronized) Duration: 4 days
Zhang et al. (2023)	Heart on chip - Micro EHTs	MODEL 4100, A-M Systems	Pt electrodes	EFV: 3–4 V/cm PD: 2 ms Freq: Varying Hz	Polarity: Biphasic Shape: Rectangular Symmetry: Symmetric	Start: After 1 day of tissue formation Duration: 7 days Frequency: Starting at 2 Hz increasing to 6 Hz with 1 Hz per day until day 7
Chan et al. (2013)	EB	C-Pace, IonOptix	Carbon electrodes	EFV: 6.6 V/cm PD: 2 ms Freq: 1 Hz	Polarity: Biphasic Shape: ND Symmetry: ND	Start After 19 days of EB formation Duration: 4 days

EFV, electric field voltage; PD, pulse duration; Freq, frequency; EHT, engineered heart tissue; ND, non disclosed.

### 3.1 Electrical field parameters

The used electrical field voltage (EFV) is designed to induce a depolarization of the CMs to mimic the natural electrical signals CM experience *in vivo*. However, *in vivo* the electrical signal inducing depolarization comes through endogenous signaling from cell-cell junctions (da Silva et al., 2020; Liu et al., 2021; Thrivikraman et al., 2018). The EFV used across different models for CM maturation varies between 2 and 6.5 V/cm.

In conjunction with EFV, the pulse duration (PD) plays a critical role in regulating the extent of ion flux across the cell membrane, which is believed to be the primary mechanism driving cardiomyocyte excitation during exogenous electrical field stimulation (da Silva et al., 2020; Thrivikraman et al., 2018). The PD must be carefully calibrated to avoid exceeding the time required for membrane depolarization, which generally is between 2 and 20 milliseconds (Thrivikraman et al., 2018). Prolonged PD beyond this window can disrupt the excitation process, potentially leading to desynchronization. In the studies reviewed, PD values typically ranged from 1 to 5 ms, thereby adhering to this principle.

Beyond ensuring effective excitation, EFV and PD should also be optimized to limit excessive charge injection, as this can promote the generation of reactive oxygen species (ROS), which may negatively impact tissue health during prolonged stimulation (Merrill et al., 2005).

### 3.2 Waveform

Another critical factor influencing ion flow is the waveform of the applied electrical pulse. Biphasic stimulation has the capacity to repolarize the cell membrane through its alternating polarity, potentially mitigating the membrane depolarization induced by initial pulse in the opposite polarity (Liu et al., 2021; Merrill et al., 2005). Additionally, biphasic pulses have been shown to reduce the generation of harmful byproducts such as reactive oxygen species *(ROS)* compared to monophasic pulses (Merrill et al., 2005). Consequently, the predominance of biphasic stimulation in most models is logical given its advantages in long-term cultures. Interestingly, in the study by Ronaldson-Bouchard et al. monophasic pulses were used, yet their study is one of the few to demonstrate several critical hallmarks like FFR, PRP, and the formation of T-tubules. This suggests that further investigation into the impact of waveform on maturation is needed. Studies investigating biphasic *versus* monophasic stimulation in 3D neonatal rat cardiac models and 3D human cardiac progenitor models have reported superior outcomes in terms of expression for cardiac markers, cardiac organization, and contraction synchronization for biphasic stimulation (Chiu et al., 2011; Gabetti et al., 2023; Pietronave et al., 2014).

When comparing results from functional tests such as ET, MCR, or FFR, it is important to recognize that different studies often use varying electrical field parameters, such as EFV, pulse duration, and waveform, to study these characteristics. These differences in experimental setup can significantly impact the absolute values reported, making direct comparisons between studies challenging.

However, many setups, particularly those utilizing the c-Pace stimulator (IonOptics), sometimes omit details regarding waveform symmetry and shape, which could provide valuable insights into the potential significance of these parameters. Optimizing these parameters could improve the threshold of excitation while simultaneously minimizing the formation of toxic byproducts such as ROS, as suggested by previous studies (Merrill et al., 2005; Nunes et al., 2013; Thrivikraman et al., 2018).

### 3.3 Electrode materials

The materials used for electrodes in direct contact with the culture medium or cells are a critical consideration when designing a setup for long-term electrical stimulation. Most studies utilize either carbon or platinum electrodes, with some employing gold-coated platinum electrodes. These materials are chosen for their reduced likelihood of producing toxic byproducts due to their favorable material characteristics (Merrill et al., 2005). Among these, carbon electrodes offer the added advantage of being more cost-effective, making them an attractive option for scalable or prolonged use.

### 3.4 Pacing protocol and timing

In the field of CM maturation, a variety of stimulation frequencies and protocols are used to achieve this goal. Table 2 highlights the range of frequencies employed, with many studies using constant physiologically relevant frequencies, such as 1–2 Hz, which approximates the adult human heart rate (60–120 bpm). Other studies utilize ramping protocols, progressively increasing the frequency from 1 Hz to 6 Hz over one to 2 weeks. Although 6 Hz is not physiological, equivalent to a heart rate of 360 bpm, these studies claim to achieve significant maturation hallmarks, such as increased desmosome formation, T-tubule formation, and a positive FFR (Nunes et al., 2013; Ronaldson-Bouchard et al., 2018). Nunes et al. further showed that ramping up to 6 Hz induced stronger maturation than ramping to 3 Hz, while Ronaldson-Bouchard et al. found that ramping to 6 Hz produced greater effects than constant pacing at 2 Hz. These findings support the suggestion by Li et al. that increased energetic demand from pacing drives mitochondrial development to sustain enhanced contractile activity.

Furthermore, electrical pacing can be initiated at different stages of differentiation. Some protocols begin early in the differentiation process (as early as 5 days after the start of differentiation), while others begin pacing after the formation of stable, spontaneously beating tissues. The timing of pacing appears to influence maturation potential, as demonstrated by Ronaldson-Bouchard et al., who reported that tissues constructed from younger CMs (12 days *in vitro*) exhibited superior maturation outcomes and expression of key hallmarks compared to tissues made from more mature CMs (28 days *in vitro*). Notably, the timing of electrical pacing might not only impact hPSC-CM maturation but may also influence subtype specification. For example, Licata et al., 2025a found that initiating stimulation earlier in embryoid body differentiation (7 days *in vitro*) favored an atrial-like phenotype, whereas later stimulation (14 days *in vitro*) promoted a more ventricular phenotype.

## 4 Future outlook

Recent studies have shown that exogenous electrical stimulation of hPSC-derived 3D cardiac tissues significantly enhances their maturation, leading to more physiologically relevant fucntional responses when compared to non-stimulated conditions. However, the precise mechanisms by which electrical stimulation promotes CMs maturation remain incompletely understood (Thrivikraman et al., 2018). A deeper investigation into the direct effects of specific electrical stimulation parameters on cardiac tissue maturation could illuminate critical pathways and processes involved. For instance, prior research comparing monophasic and biphasic stimulation has underscored the importance of these parameters; however, contradictory findings, such as those reported by Ronaldson-Bouchard et al., highlight the current limitations in understanding (Chiu et al., 2011; Gabetti et al., 2023; Pietronave et al., 2014). Further elucidation of electrical parameters, including waveform characteristics, will be essential for advancing the field.

Moreover, the role of reactive oxygen species (ROS) in cardiac tissue maturation requires additional research, particularly comparing various methods for excitation stimulation, including ones without ROS production. A parallel method for excitation stimulation involves the use of channelrhodopsins, which enables CM excitation via optogenetic light stimulation rather than exogenous electrical field application (Gruber et al., 2022). Optogenetic stimulation has already shown signs of tissue maturation in primary human myoblast tissues (Mills et al., 2019). Another emerging technology is photovoltaic stimulation, where light is converted into localized electrical cues through embedded micro-solar elements, enabling cardiomyocyte excitation without conventional electrode setups (Ershad et al., 2025). By reducing or eliminating charge injection, the primary source of stimulation-induced ROS (Merrill et al., 2005), such methods allow more controlled studies on how excitation contributes to CM maturation, independent of ROS byproducts.

The integration of 3D cultures with exogenous electrical stimulation already represents a multifaceted approach for promoting CM maturation. However, future advancements are likely to arise from incorporating additional maturation methods. For example, the inclusion of multiple supporting cell types besides fibroblasts, such as hPSC-derived macrophages, has been shown to enhance maturation under electrical pacing (Lock et al., 2024). Their role in cardiac tissue maturation is to clear out apoptotic and mitochondrial debris, which reduces stress on the microtissue and enhances the development and function of the contractile machinery in CMs (Hamidzada et al., 2024). This raises the possibility that incorporating macrophages could help mitigate some of the stress responses associated with electrical stimulation. Furthermore, mechanical stimulation has demonstrated synergistic effects when combined with electrical stimulation, leading to further improvements in tissue maturation (Ahmed et al., 2020; Ruan et al., 2016). Other external stimuli, including hypoxia, perfusion, and optimized culture media, have also been employed to enhance cardiac maturation (Stein et al., 2021; Stoppel et al., 2016). These approaches, when combined with electrical and mechanical stimulation, hold promise for advancing the maturation of 3D hPSC-derived cardiac tissues.

The future applications of these more mature 3D hPSC-derived cardiac tissues lie in (high-throughput) toxicity and drug screening platforms as well as cardiac disease modelling. Emerging technologies, such as the MilliPore system (Tamargo et al., 2021), high-throughput Biowire (Sun and Nunes, 2016), and miniaturized EHTs (Windt et al., 2023), exemplify the trend toward miniaturized platforms that require fewer resources while enabling high-throughput screening. Notably, this miniaturization also impacts the mechanisms of electrical stimulation, reducing the formation of toxic byproducts and ROS, thereby enhancing the safety and effectiveness of these platforms for drug discovery and development (Merrill et al., 2005). The further rise of commercially available platforms for the use of electrical stimulation on cells shows the viability and future potential of this domain. With platforms such as Cuore (Optics11), C-Dish (IonOptics), and µBeat (BioMimix) research into the effects of electrical stimulation on cells becomes more available.

In summary, electrical stimulation significantly enhances the maturation of hPSC-CM structures, promoting physiological development and improving key maturation hallmarks such as conduction velocity, action potential characteristics, and calcium handling. Although the focus on cardiac tissue engineering often centers on biological aspects, the electrical engineering and design of stimulation systems are crucial for ensuring reproducible and optimal maturation outcomes. As we advance, further research is essential to optimize pacing protocols, particularly in fine-tuning timing parameters to better mimic physiological conditions and maximize tissue functionality. Continued investigation into both biological and technological parameters will be essential for bridging the gap between experimental *in vitro* models and their translational applications, including drug testing and disease modelling.
